# Plasma‐Induced 2D Electron Transport at Hetero‐Phase Titanium Oxide Interface

**DOI:** 10.1002/advs.202304919

**Published:** 2023-12-07

**Authors:** Kehan Yu, Xinglong Li, Haoyu Zhao, Chen Ma, Zhongyue Wang, Peng Lv, Ertao Hu, Jiajin Zheng, Wei Wei, Kostya (Ken) Ostrikov

**Affiliations:** ^1^ College of Electronic and Optical Engineering & College of Flexible Electronics (Future Technology) Nanjing University of Posts and Telecommunications Nanjing 210023 China; ^2^ Jiangsu Province Engineering Research Center for Fabrication and Application of Special Optical Fiber Materials and Devices Nanjing 210036 China; ^3^ School of Chemistry and Physics and QUT Centre for Materials Science Queensland University of Technology (QUT) Brisbane QLD 4000 Australia

**Keywords:** electron transport, heterointerfaces, layered materials, plasma nanotechnology, titanium dioxide

## Abstract

Interfaces of metal oxide heterojunctions display a variety of intriguing physical properties that enable novel applications in spintronics, quantum information, neuromorphic computing, and high‐temperature superconductivity. One such LaAlO_3_/SrTiO_3_ (LAO/STO) heterojunction hosts a 2D electron liquid (2DEL) presenting remarkable 2D superconductivity and magnetism. However, these remarkable properties emerge only at very low temperatures, while the heterostructure fabrication is challenging even at the laboratory scale, thus impeding practical applications. Here, a novel plasma‐enabled fabrication concept is presented to develop the TiO_2_/Ti_3_O_4_ hetero‐phase bilayer with a 2DEL that exhibits features of a weakly localized Fermi liquid even at room temperature. The hetero‐phase bilayer is fabricated by applying a rapid plasma‐induced phase transition that transforms a specific portion of anatase TiO_2_ thin film into vacancy‐prone Ti_3_O_4_ in seconds. The underlying mechanism relies on the screening effect of the achieved high‐density electron liquid that suppresses the electron‐phonon interactions. The achieved “adiabatic” electron transport in the hetero‐phase bilayer offers strong potential for low‐loss electric or plasmonic circuits and hot electron harvesting and utilization. These findings open new horizons for fabricating diverse multifunctional metal oxide heterostructures as an innovative platform for emerging clean energy, integrated photonics, spintronics, and quantum information technologies.

## Graphical abstract

A new non‐thermal plasma method is used to fabricate a TiO_2_/Ti_3_O_4_ hetero‐phase bilayer in just seconds. The high‐concentration electron gas at the heterointerface condenses into 2D electron liquid (2DEL) at room temperature. The 2DEL shields the scattering between electrons and phonons, allowing electrons to flow “adiabatically”. These features are suitable for low‐loss electronics or plasmonics and hot electron utilization.

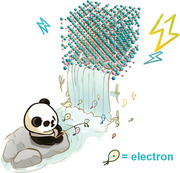

## 1 Introduction

Metal oxide heterointerfaces exhibit rich electron correlation phenomena,^[^ ^1^ ^]^ including superconductivity,^[^ ^2^ ^]^ metal‐insulator transitions,^[^ ^3^ ^]^ Mott insulators,^[^ ^4^ ^]^ colossal magnetoresistance,^[^ ^5^ ^]^ and 2D electron liquids (2DELs).^[^ ^6^ ^]^ However, many of these exotic properties can only be observed at very low temperatures. Consequently, despite decades of intense global efforts, these heterointerfaces have not yet been utilized in practical applications. These interfaces are typically fabricated by heteroepitaxial growth processes that require precise control of the structure and the electronic properties using complex and costly methods such as molecular beam epitaxy and pulsed laser deposition. Moreover, it often appears challenging to achieve the desired materials combinations while ensuring the heterointerface quality and stability. For instance, the creation of 2DELs in LaAlO_3_/SrTiO_3_ (LAO/STO) heterostructures necessitates the TiO_2_/LaO‐terminated interface, which is difficult to produce in a reproducible and scalable way. Above all, very low temperatures are still inevitable to achieve the 2D superconductivity and magnetism for the 2DEL in LAO/STO systems.

Therefore, new alternative approaches are vitally needed to create oxide interfaces that (i) are effective, that is, sustain 2D electron systems with “quantum” properties, and (ii) can be produced in a simple and cheap process, thereby alleviating the stringent epitaxy requirements that constrain device miniaturization across several industry sectors. Solving these problems will allow more flexibility for novel oxide electronics platforms for quantum information, neuromorphic computing, spintronics, high‐temperature superconductivity, and other emerging technologies.^[^ ^7^ ^]^

Here, we propose a conceptually new innovative solution based on low‐temperature plasmas that, in one rapid and simple step, generates a 2DEL at a self‐formed titanium oxide heterointerface featuring room‐temperature weakly localized Fermi liquid, while simultaneously eliminating the seemingly unavoidable need for epitaxial growth. In our solution, we exploit the versatility and environmentally friendliness of the non‐equilibrium low‐temperature plasmas, which offer unique advantages in the deposition, etching, and modification of materials for applications in nanoelectronics, energy conversion and storage, nanomaterials, surface engineering, and other fields.^[^ ^8^ ^]^

We further utilize the key feature of low‐temperature plasmas to selectively modify the surface of solids, and recent discoveries of the plasma's ability to create unconventional phases in metal oxide nanomaterials, which are otherwise unattainable by conventional methods.^[^ ^9^ ^]^ Our hypothesis is that the plasma‐induced phase transition (PIPT) may facilitate the heterojunction formation between distinct phases, leading to the enhanced charge transfer and mass transport properties of the nanomaterials.^[^ ^9a^ ^]^

The key feature of our approach is the effective and rapid (within seconds) creation of a TiO_2_/Ti_3_O_4_ heterointerface, where the low‐temperature plasma enables inward growth with crystallographic alignment and intentional vacancy defect creation in Ti_3_O_4_. The underlying mechanism is based on the difference in chemical potential between Ti_3_O_4_ and TiO_2_ which causes the interfacial electron transfer, resulting in a 2DEL with the electron density (10^14^ cm^−2^ or ≈10^21^ cm^−3^) an order of magnitude higher than that in the LAO/STO. The high‐density electrons screen the electron–phonon (*e–ph*) interactions, thereby enabling weakly localized Fermi liquid features at room temperature. The strong electron–electron (*e–e*) interactions make the electrons appear to transport “adiabatically” in the 2DEL, which is highly desired in low‐loss electric or plasmonic circuits and hot electron harvesting and utilization. These findings are important advances in the fabrication of oxide heterointerfaces for applications across diverse fields ranging from nanoelectronics and spintronics to quantum information.

## 2 Results and Discussion

### 2.1 Fabrication and Structural Characterization of TiO_2_/Ti_3_O_4_

**Figure 1:**
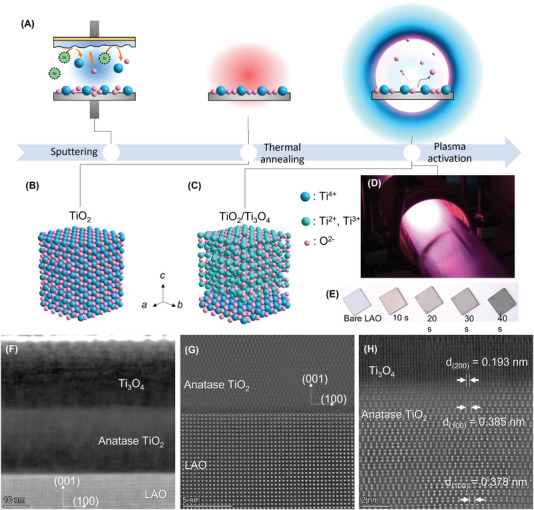
Plasma‐enabled fabrication and microstructure of the TiO_2_/Ti_3_O_4_ hetero‐phase bilayer. A) Schematics of fabrication of TiO_2_/Ti_3_O_4_ hetero‐phase bilayer, including sputtering, thermal annealing, and plasma activation in sequence. The TiO_2_ was in the anatase phase after thermal annealing B) and was converted to TiO_2_/Ti_3_O_4_ hetero‐phase bilayer after the plasma activation (C). D) Photograph of Ar/H_2_ plasma glow ball used for PIPT. E) Photographs of bare LAO substrate and TiO_2_/Ti_3_O_4_ on LAO with 10–40 s plasma processing. F) Cross‐sectional HAADF STEM image of the bilayer, and close views of G) LAO/TiO_2_ and H) TiO_2_/Ti_3_O_4_ interfaces.

The TiO_2_/Ti_3_O_4_ hetero‐phase bilayer was fabricated by thermal annealing a sputtered epitaxial TiO_2_ thin film deposited on LAO followed by the low‐temperature plasma activation, as schematically shown in **Figure** **1**A. The as‐deposited TiO_2_ thin film on LAO (001) plane was initially amorphous, but underwent crystallization to anatase TiO_2_ film after post‐thermal annealing. The surface of the as‐deposited TiO_2_ thin film was ultra‐smooth, and became slightly rough after annealing, as confirmed by the atomic force microscopy images in Figure S1 (Supporting Information). After a 10‐s plasma treatment, a portion of the anatase TiO_2_ layer was converted to Ti_3_O_4_, as illustrated in Figure 1B,C. Since TiO_2_ and some metal oxides (e.g., ZnO, CdO, In_2_O_3_, WO_3_, and MoO_3_) are usually deficient in O in equilibrium, the chemical potential of electrons can be elevated by reducing the O content in the oxides. Upon brief plasma exposure, some oxygen atoms close to the outermost surface layer are released, and a vacancy‐prone TiO_2_/TiO_2‐_ *_δ_* heterojunction can be formed.

In the PIPT process, a dazzling glow ball and a small amount of hydrogen are essential: the former generates localized high‐density energy (Figure 1D), and the latter stabilizes the Ti_3_O_4_ at ambient conditions. This approach contrasts sharply with conventional heat treatment and diffuse plasmas, which take hours and transform the entire TiO_2_ to a defect‐rich form (known as black TiO_2_).^[^ ^10^ ^]^ The films after the plasma treatment appear gray and darken with longer treatment time (10–40 s, Figure 1E), which resembles the previously reported black TiO_2_ with a large number of oxygen vacancies and more pronounced structural disorder.^[^ ^10^ ^]^ The dark appearance of the films indicates the Drude absorption in the visible and near‐infrared regions by free electrons. The H atoms can occupy the oxygen vacancies in the Ti_3_O_4_ then prevent its oxidization, making the heterostructure stable over months. The H atoms can form bonding with the Ti_3_O_4_, just as previously reported for hydrogenated TiO_2_.^[^ ^10^, ^11^ ^]^ The mobility of one sample was originally 12.7 ± 1.3 cm^2^ V^−1^ s^−1^, then it remained almost unchanged (14.8 ± 1.9 cm^2^ V^−1^ s^−1^) 9 months after its production. Without hydrogen, the as‐prepared TiO_2_/Ti_3_O_4_ hetero‐phase bilayer would be oxidized instantly upon contact with air. This will appear as fading of color and loss of electric conductance of the film. Further discussion is provided in Supporting Information. The overall production of heterostructures by PIPT is less costly than the conventional molecular‐beam epitaxy and pulsed laser deposition, since no ultra‐high vacuum, expensive equipment, additional laser sources, or expensive gases such as ArF and KrF are needed. It is worth mentioning that the PIPT method can be easily scaled up using commercial inductively‐coupledplasma (ICP) devices, which can process wafer‐size substrates. The advances in ICP equipment would ultimately limit the scaling up of the PIPT.

The microstructure and interface of the bilayer were characterized using scanning transmission electron microscopy (STEM). The cross‐sectional high‐angle annular dark field (HAADF) STEM images confirmed the lamination, crystallinity, and crystallographic alignment of each layer (Figure 1F–H). The observed total thickness of the titanium oxide bilayer is ≈50 nm, which agrees with the thickness of the sputtered layer. After the short plasma treatment, the top half of the thin film (i.e., 25 nm in thickness, Figure 1F) was converted to the Ti_3_O_4_ phase. Meanwhile, the lower layer of the TiO_2_ film retained the anatase structure. The anatase TiO_2_ was formed on the (001) plane of the LAO with a perfectly coherent and smooth interface (Figure 1G). The Ti_3_O_4_ layer activated by the plasma exhibits predominantly vertical lattice fringes and a similar lattice constant with the underneath anatase TiO_2_ (Figure 1H). The Ti_3_O_4_ layer has an inter‐plane spacing between (200) planes *d*_(200)_ = 0.193 nm, which is slightly larger than half of the *d*_(100)_ of the anatase TiO_2_ (Figure 1H). More discussion on the strains in the hetero‐phase bilayer can be found in Supporting Information.

**Figure 2:**
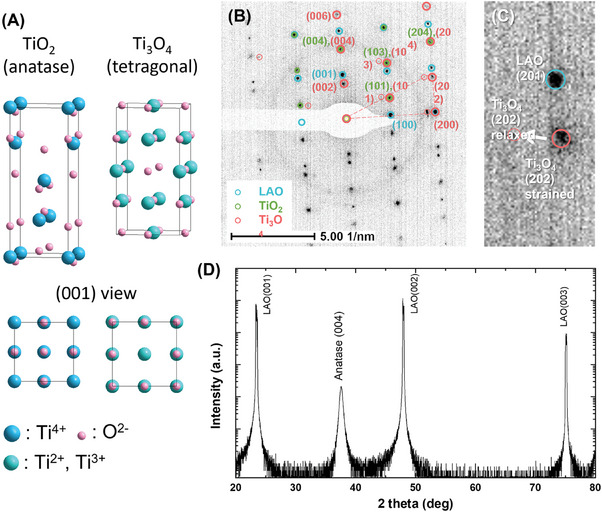
Crystallography of the TiO_2_/Ti_3_O_4_ hetero‐phase bilayer. A) The unit cells of anatase TiO_2_ and tetragonal Ti_3_O_4_, and their views along [001] axis. The structures are constructed using the data in the Materials Project.^[^ ^12^ ^]^ B) SAED pattern taken from the area shown in Figure 1F. Diffraction spots from the LAO substrate, TiO_2_, and Ti_3_O_4_ films are differentiated by colored circles and indices. C) The strained and partially relaxed (202) diffraction of Ti_3_O_4_. D) HRXRD pattern of the TiO_2_/Ti_3_O_4_ hetero‐phase bilayer.

The Ti_3_O_4_ is a tetragonal structure that is predicted by the Materials Project and has not been discovered in experiments before. The Ti_3_O_4_ crystallizes in the tetragonal I4/mmm space group and has *a* = *b* = 0.413 nm and *c* = 0.818 nm in theory,^[^ ^12^ ^]^ very close to that of the anatase TiO_2_ (shown in **Figure** **2**A). This enables the potential coherent growth of Ti_3_O_4_ on TiO_2_. Selected area electron diffraction (SAED) patterns taken from the cross section of the bilayer, i.e., the area shown in Figure 1F, is shown in Figure 2B. The diffraction spots can match the predicted very well, which verifies the formation of tetragonal Ti_3_O_4_. A comparison between the measured and predicted diffraction patterns can be found in the Supporting Information.

It is worth noting that the strains in the Ti_3_O_4_ provide another evidence of coherent growth. If the TiO_2_ and Ti_3_O_4_ layers have a perfectly coherent interface, the (200) spots of them should completely overlap, and the Ti_3_O_4_ is under strain. On the other hand, the Ti_3_O_4_ is partially relaxed as revealed by lighter satellite spots around (200), (202), (101), (103), and so on (Figure 2B). A close examination of the (202) diffraction indicates that the Ti_3_O_4_ is primarily under strain while is just slightly relaxed (Figure 2C). Converted from the diffraction spots, the lattice constant of Ti_3_O_4_, *d*_(200)_ = 0.187 nm, which is consistent with the STEM data (0.193 nm). The partially relaxed lattice constant of Ti_3_O_4_, *d*_(200)_ = 0.205 nm, agrees well with the predicted one (0.206 nm). These results indicate that the Ti_3_O_4_ is under compressive stress along the *a* and *b* axes, and under tensile stress along the *c* axis, which is exactly observed in the STEM.

The TiO_2_/Ti_3_O_4_ hetero‐phase bilayer was further investigated by high‐resolution XRD (HRXRD). As reported by the HRXRD *ω*–2𝜃 scan in Figure 2D, the diffraction pattern displays only the peaks of LAO and (004) of anatase TiO_2_. The (004) peak of Ti_3_O_4_ cannot be distinguished since it overlaps that of TiO_2_. Moreover, the (006) of Ti_3_O_4_ is also undetectable, as the diffraction intensity of (006) is 2 orders of magnitude weaker than that of (004), as proposed by the Materials Project. The exclusive (004) peak of TiO_2_ in the HRXRD pattern reveals that the TiO_2_ is well aligned with LAO substrate in the [00*l*] direction, which is a good indication for epitaxial growth. The thickness of the TiO_2_ layer can be estimated based on the broadening of its (004) diffraction peak, which is 0.45°. Through Scherrer equation, the thickness is calculated to be 19.47 nm, which is close to that observed by STEM. The thickness of the film can also be derived from the X‐ray reflection (XRR) simulation of the experimental curve. Based on the measured and fitted XRR results, the thicknesses of TiO_2_ and Ti_3_O_4_ are 28 and 27 nm, respectively. More discussion on the film thickness and uniformity can be found in Supporting Information.

Defects in Ti_3_O_4_ are critical for 2DELs because the conductive crystalline Ti_3_O_4_ (zero bandgap) would short‐circuit the electron conduction in 2DEL. The weak diffraction intensity and dull appearance of the Ti_3_O_4_ layer in the STEM images (Figures 1H and 2C) might indicate a lower density caused by the abundant defects. Although Ti_3_O_4_ has a higher packing density than TiO_2_ in theory, its thickness does not vary after the plasma treatment, suggesting the presence of some internal voids.

### 2.2 Electron Transfer Across the Heterointerface

**Figure 3:**
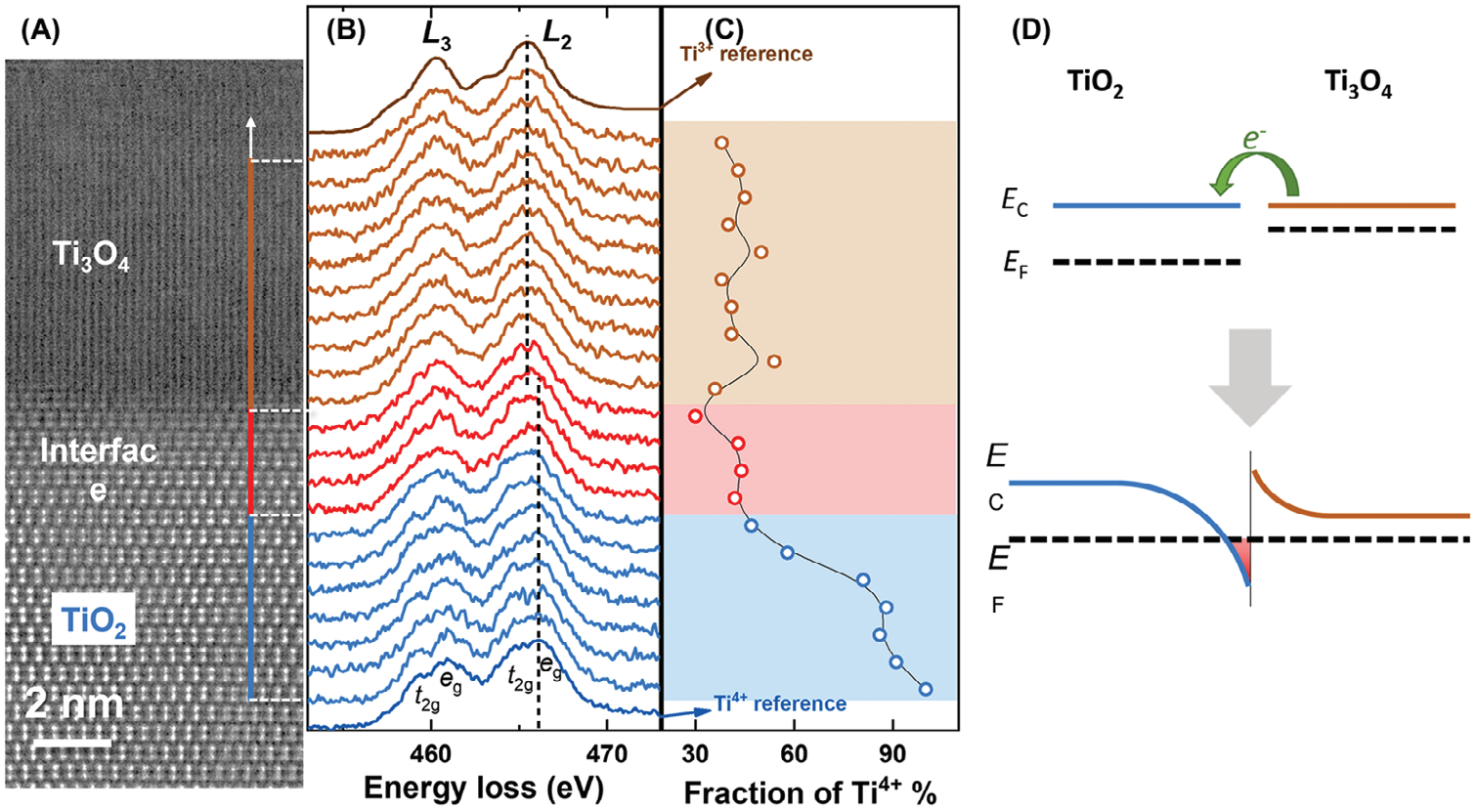
Electron transfer across the TiO_2_/Ti_3_O_4_ heterointerface caused by the created difference in chemical potential. A) An EELS line scan conducted across the interface with a point interval of ≈0.75 nm. B) The recorded ELNES of the Ti‐*L* edge at every point. Reference Ti‐*L* edges of corundum Ti_2_O_3_ and anatase TiO_2_ are placed on top and bottom, respectively. C) Proportion of Ti^4+^ at each point. D) Schematic illustrations of electron level configurations and band bending across the heterointerface.

The occurrence of electron transfer at the TiO_2_/Ti_3_O_4_ interface may result in a reduction of Ti^4+^ charging state in the vicinity of the interface. To investigate the microscopic distribution of valence states near the heterointerface, we performed an electron energy loss spectroscopy (EELS) line scan in STEM. **Figure** **3**A shows a line segment of ≈15 nm across the interface along the [001] direction of anatase TiO_2_. The energy‐loss near‐edge structures (ELNES) of the Ti‐*L* edge at every point with an interval of ≈0.75 nm were acquired. Deep in the anatase TiO_2_, the Ti‐*L*_2_ and *L*_3_ edges exhibit clear *t*_2g_ and *e*_g_ sub‐band splitting, but this splitting becomes less distinct within ≈3 nm from the interface (Figure 3B). Whereas in Ti_3_O_4_ above the interface, the ELNES is almost featureless (Figure 3B). For better comparison, reference Ti‐*L* edges of corundum Ti_2_O_3_ and anatase TiO_2_ are also displayed in Figure 3B. Considering the quite similar EELS spectra for Ti^2+^ and Ti^3+^, especially at the Ti‐*L*_2,3_ edge, it is reasonable to take the Ti_2_O_3_ as a reference.^[^ ^13^ ^]^ Moreover, the fraction of Ti^4+^ ions at various positions in the film obtained by linear combination and linear least‐square fitting is shown in Figure 3C. Deep in the TiO_2_ (brown area in Figure 3C) and Ti_3_O_4_ layers (blue area in Figure 3C), the proportions of Ti^4+^ are ≈90% and 40%, respectively. In the interface layer (red area in Figure 3C), the proportion of Ti^4+^ is ≈40%, indicating the reduction effect by electrons transferred from the Ti_3_O_4_ layer. It is worth noting that the drastic change in the Ti^4+^ ratio in the ±1 nm region on both sides of the interface indicates the depletion at the Ti_3_O_4_ side (54% of Ti^4+^) and accumulation at the TiO_2_ side (30% of Ti^4+^).

The reduction of Ti^4+^ and the removal of oxygen from TiO_2_ lead to a higher electron concentration, which elevates the chemical potential (i.e., Fermi level *E*_F_) and lowers the work function. Consequently, at the TiO_2_/Ti_3_O_4_ interface, electron transfer to TiO_2_ takes place, enabling the formation of 2DEL on the TiO_2_ side (Figure 3D). The work function of Ti_3_O_4_ was measured by ultraviolet photoelectron spectroscopy (UPS) and found to be 4.12 eV (Figure S2, Supporting Information), which is 0.6 eV lower than the reported work function of anatase TiO_2_.^[^ ^14^ ^]^ The higher chemical potential in the Ti_3_O_4_ creates favorable conditions for the cross‐interface electron transfer from Ti_3_O_4_ to TiO_2_.

### 2.3 Electron Transport Properties of TiO_2_/Ti_3_O_4_ Hetero‐Phase Bilayer

**Figure 4:**
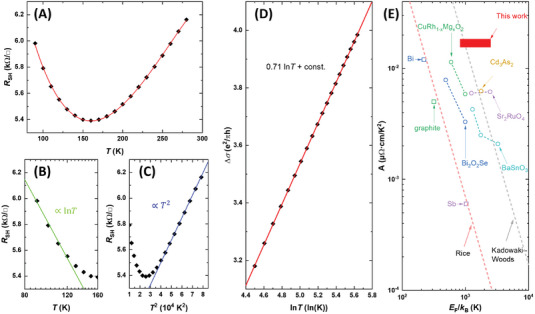
Weakly localized Fermi liquid features in the TiO_2_/Ti_3_O_4_ hetero‐phase bilayer. A) Temperature‐dependent *R*_SH_, B) ln*T* fitting below 160 K, and C) *T* ^2^ fitting above 160 K. D) The correction to the conductivity of a 2D Fermi liquid system linearly fitted with ln*T* expressed in quantum conductance ($\frac{e^{2}}{\pi h}$). E) *A*  versus  *E*_F_ plot for various Fermi liquids, including Bi,^[^ ^17^ ^]^ Sb,^[^ ^17^ ^]^ graphite,^[^ ^18^ ^]^ Cd_3_As_2_,^[^ ^19^ ^]^ Sr_2_RuO_4_,^[^ ^20^ ^]^ Bi_2_O_2_Se,^[^ ^21^ ^]^ BaSnO_3_,^[^ ^22^ ^]^ and CuRh_1‐x_Mg_x_O.^[^ ^23^ ^]^ The approximate position of the TiO_2_/Ti_3_O_4_ hetero‐phase bilayer is represented by the red bar. The Kadowaki–Woods and Rice scaling relations are plotted using dash lines.

The TiO_2_/Ti_3_O_4_ hetero‐phase bilayer displays a 2DEL transport which appears similar to a typical Fermi liquid in the weak localization regime. As shown in **Figure** **4**A, the rise and fall trends of the sheet resistance (*R*_SH_) with temperature are divided at 160 K. Appropriate fittings reveal that the temperature dependence of the *R*_SH_ shows a logarithmic decrease from 90 to 150 K (Figure 4B) and a quadratic increase above 160 K. (Figure 4C). The resistance upturn with ln*T* below 150 K could be attributed to *e–e* interaction in the weak localization regime, while the ln*T* dependence is only relevant for 2D electron systems.^[^ ^15^ ^]^ The *T* ^2^ dependence above 160 K also suggests that the dominant scattering mechanism is the *e–e* interaction, which is consistent with the behavior of a Fermi liquid system. Below the Debye temperature (≈700 K for anatase TiO_2_^[^ ^16^ ^]^), the electron–phonon (*e–ph*) scattering would normally cause the resistance to vary following the ≈*T* ^5^ trend. Considering all the contributions from *e–e* and *e–ph* interactions, the overall resistance variation versus temperature can be described by the following equation:

(1) $$R_{SH}=R_{0}\;+R_{1}T^{2}+R_{2}T^{5}+R_{3}\ln\frac{1}{T}$$

where *R*_0_ is the residual resistance, *R*_1_, *R*_2_, and *R*_3_ are the fitting factors. The resulting fitting curve is shown in Figure 4A (red curve), and fitting parameters are listed in Table S2 (Supporting Information). The fitting confidence is also discussed in Supporting Information. The negligibly small *R*_2_ suggests that the *e–ph* interaction is almost missing in the 2DEL.

On one hand, one can formulate the deviation of the resistance from a Fermi liquid system as follows.

(2) $$\Delta R\;=R_{\text{SH}}\;-\left(R_{0}+R_{1}T^{2}+R_{2}T^{5}\right)$$

The correction to the corresponding conductivity expressed in quantum conductance ($\frac{e^{2}}{\pi h}$) is

(3) $$\Delta \sigma \;=\;\Delta \;\left(\frac{1}{R}\right)=\;-\left(\frac{\Delta R}{R_{0}^{2}}\right)/\left(\frac{e^{2}}{\pi h}\right)$$

where *e* and *h* are the elementary charge and the Planck constant, respectively. Figure 4D shows that ∆𝜎 has a linear relationship with ln*T*, with a slope value of 0.71. This indicates that a quantum correction mechanism is involved, as ∆𝜎 is related to the quantum conductance. Furthermore, Δσ∝ln*T* is also a typical observation for electron coherent backscattering in 2D systems.^[^ ^24^ ^]^ The slope value less than 1 could also be attributed to the effect of *e–e* interactions.^[^ ^24a^ ^]^ Based on the consideration of transport theory, experimental data, and different transport mechanisms between the heterostructure and pure Ti_3_O_4_, it is concluded that the 2D transport is indeed the dominant transport mechanism in the TiO_2_/Ti_3_O_4_ hetero‐phase bilayer. A comprehensive discussion on the determination of 2D transport is provided in Supporting Information.

On the other hand, in Fermi liquids (e.g., metals, semimetals, dilute metals, and doped semiconductors), the relationship between resistivity *ρ* and *T* is expressed as *ρ* = *ρ*_0_ + *A T* ^2^ at sufficiently low temperatures due to *e–e* interactions, where *ρ*_0_ is the residual resistivity and *A* is a prefactor.^[^ ^25^ ^]^ Previous studies have found that the *A* correlates with Fermi energy *E*_F_ following either the Kadowaki–Woods (K–W) scaling for strongly correlated materials, or the Rice scaling for weakly correlated materials (dash lines in Figure 4E).^[^ ^26^ ^]^ As shown in Figure 4E, semimetals (Bi, graphite, Sb, and Cd_3_As_2_) roughly follow the Rice scaling, while the strongly correlated metals, dilute metals, and doped semiconductors (Sr_2_RuO_4_, Bi_2_O_2_Se, BaSnO_3_, and CuRh_1‐x_Mg_x_O) typically follow the K–W scaling. The *A* of our hetero‐phase bilayer, that is, *R*_1_ in Equation 1 multiplied by the thickness of the 2DEL, falls into the K–W regime (red bar in Figure 4E), indicating the presence of strongly correlated electrons in our case.

Generally speaking, the *T* ^2^ dependence of resistivity only occurs at low temperatures because *e–ph* scattering disrupts *e–e* interactions as the temperature rises. However, the observed *T* ^2^ dependence in our study occurring from 160 K to room temperature is highly atypical. Given that the prefactor *R*_2_ of the *T* ^5^ term is negligible (Table S2, Supporting Information), this suggests an unexpectedly weak *e–ph* interaction, which is a rare phenomenon in electron transport in solids. The Fermi liquid at room temperature may be due to the high concentration of electrons that screens the *e–ph* coupling (i.e., polarons). The characteristic time of electron screening can be estimated by the frequency of the collective oscillation of conduction electrons in solids (i.e., plasmon frequency) ω_p_ = (*ne* ^2^/ε_0_ε_∞_ m*)^1/2^ , where *n* is the electron concentration in the solid material, ε_0_ the permittivity of vacuum, ε_∞_ the high‐frequency dielectric constant of TiO_2_, and m* the effective mass of electrons.^[^ ^27^ ^]^ When the electron concentration is high enough, the plasmon oscillations can be as fast as the phonon oscillations characterized by the frequency ω_ph_. Recent calculations showed that the corresponding electron concentration is 1 × 10^20^ cm^−3^ for anatase TiO_2_, which is consistent with the result of this work (3 × 10^20^ cm^−3^).^[^ ^27^ ^]^ Previous studies have also pointed out that *e–ph* screening is enhanced in low‐dimensional structures.^[^ ^28^ ^]^ In short, high‐concentration interfacial electrons in 2D structure should cause the ≈*T* ^2^ dependence.

**Figure 5:**
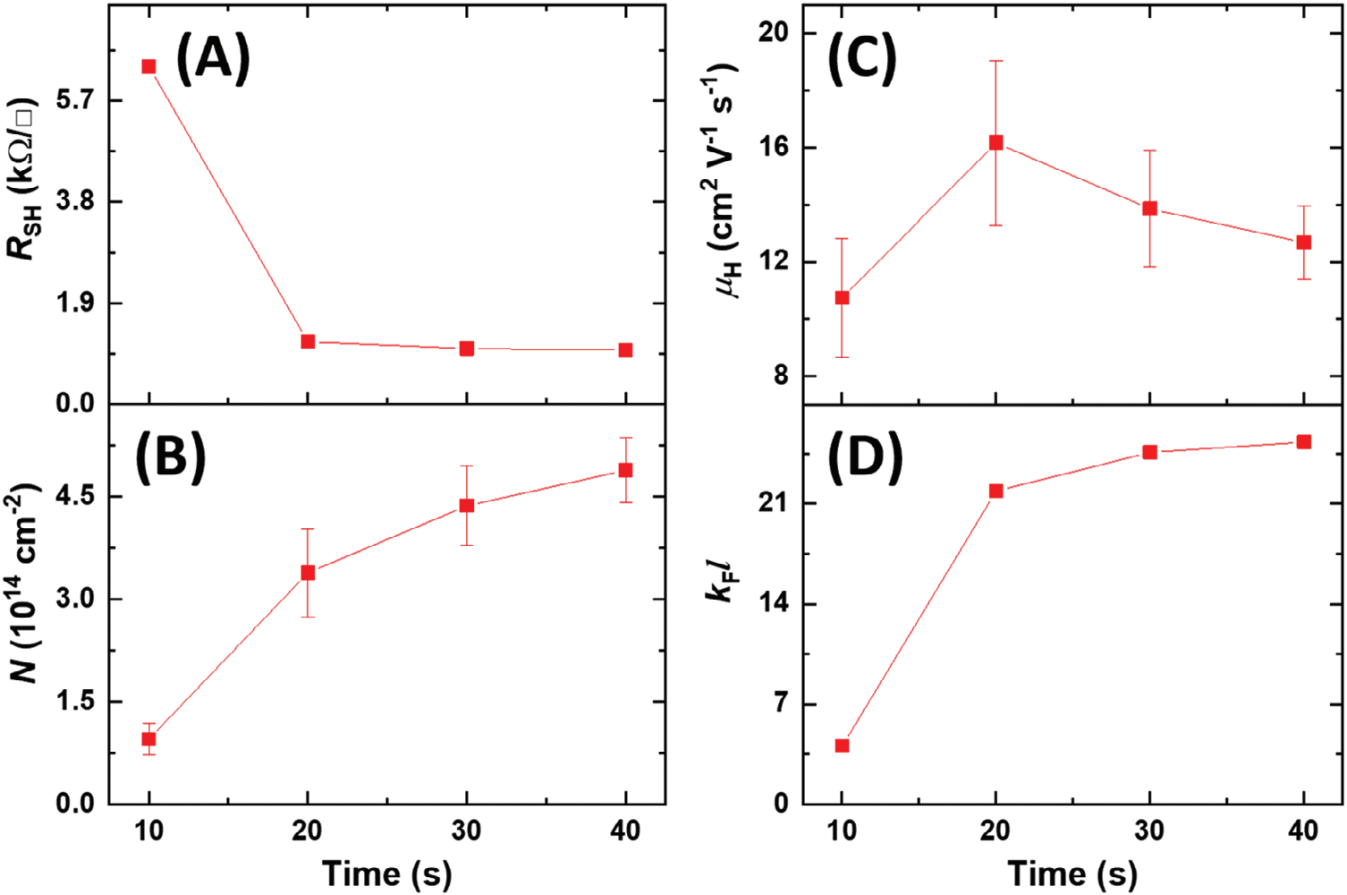
Tunable electron transport properties in the TiO_2_/Ti_3_O_4_ hetero‐phase bilayer. The variation of A) sheet resistance, B) electron concentration, C) Hall mobility, and D) Ioffe‐Regel criterion factor *k*_F_ *l* obtained for plasma treatment from 10 to 40 s.

If the properties of 2DELs are tunable, then it may be possible to obtain Fermi liquids at higher temperatures (beyond room temperature), thus making the relevant applications (will be discussed below) more temperature tolerant. As shown in **Figure** **5**, the electron transport properties of the hetero‐phase structures are influenced by the duration of the plasma processing. The *R*_SH_ dropped sharply from 6 to ≈1 kΩ/□ with increasing the treatment time and then became stable (Figure 5A). The electron concentration (*N*) increased steadily from 0.9 × 10^14^ to ≈5 × 10^14^ cm^−2^ (Figure 5B). The Hall mobility (*µ*_H_) remained relatively constant, reaching up to 16 cm^2^ V^−1^ s^−1^ (Figure 5C). These changes can be attributed to the creation of more oxygen vacancies in Ti_3_O_4_ by the prolonged plasma treatment, which raised the *E*_F_ and consequently increased *N* while reducing *R*_SH_. However, when the plasma treatment time exceeded 40 s, *µ*_H_ dropped abruptly below the instrument detection limit (1 cm^2^ V^−1^ s^−1^). This may be due to the complete transformation of TiO_2_ to Ti_3_O_4_, which resulted in very low mobility owing to the highly defective structure.

According to the Ioffe‐Regel criterion, electron transport is weakly localized when the product of the Fermi wave number (*k*_F_) and the mean free path (*l*) is much larger than one (*k*_F_ *l* >> 1). For a 2D transport, one can show that

(4) $$k_{F}l\;=\frac{h}{e^{2}}\;\;{\left(R_{\mathrm{SH}}\right)}^{-1}=\frac{25.8\;k\Omega }{R_{\mathrm{SH}}}\;$$

With *R*_SH_ = 6.34 kΩ/□ at room temperature, *k*_F_ *l* equals 4.07, just indicating a weakly localized 2D transport in the hetero‐phase bilayer. The plasma treatment time also affects the *k*_F_ *l* value and the mean free path *l* of the electrons. As shown in Figure 5D, the *k*_F_ *l* increases with the treatment time, which corresponds to the decrease of *R*_SH_. The *l* varies from ≈2 to > 4 nm, and it follows the trend exactly as *µ*_H_ does (Figure S3, Supporting Information). The calculation of the mean free path *l* can be found in Supporting Information. We attribute the increase of *l* to the enhanced screening effect as the electron concentration increases.

### 2.4 Potential Applications of TiO_2_/Ti_3_O_4_ Hetero‐Phase Bilayer

The room‐temperature Fermi liquid characteristics of our hetero‐phase bilayer may be highly suitable for hot electron collection in photovoltaic renewable energy conversion and utilization. In most inorganic semiconductor materials, hot electrons with energies above the semiconductor bandgap collide with phonons and rapidly (in sub‐ps) relax to the band edge, causing energy above the semiconductor bandgap in solar photons to be dissipated as heat. This thermalization loss accounts for 35% of the available solar energy.^[^ ^28a^ ^]^ Utilizing materials with weak *e–ph* interactions to effectively collect hot electrons has the potential to surpass the Shockley–Queisser limit for photovoltaic devices, increasing the theoretical efficiency to 66%.^[^ ^28a^ ^]^

In addition, low‐loss electron transport is a highly desirable property for future integrated photonics and quantum computing. Extremely low loss can enable the materials to have a refractive index close to zero at a specific wavelength, namely near‐zero‐index (NZI) materials. Research has demonstrated that NZI material can significantly enhance the depth and rate of optical modulation, the extremely high optical nonlinearity coefficient, leading to the ultra‐long coherence length.^[^ ^29^ ^]^ Based on these properties, ultra‐fast optical switches, ternary logic, remote quantum entanglement waveguides, and quantum emitters have received increasing attention.

## 3 Conclusion

To overcome difficulties in the epitaxy‐dominated fabrication of oxide heterostructures with pronounced quantum properties at room temperatures, we have successfully fabricated a TiO_2_/Ti_3_O_4_ hetero‐phase bilayer using an innovative PIPT within a matter of seconds. The coherent interface between the TiO_2_ and Ti_3_O_4_ phases was identified using STEM. Electron transfer from the Ti_3_O_4_ to the TiO_2_ phase, resulting in a 2DEL at the heterointerface, was detected using EELS. The thus formed 2DEL exhibited electron mobility up to 16 cm^2^ V^−1^ s^−1^, electron density as high as 4 × 10^14^ cm^−2^, and thickness of ≈3 nm. The sheet resistance and electron density can be modulated by adjusting the duration of the plasma treatment. The transport characteristics of the 2DEL resemble that of a room‐temperature Fermi liquid with quantum interference correction, indicating strong *e–e* interactions and weakly localized electron transport. The unexpectedly revealed negligible *e–ph* interaction in the 2DEL makes the hetero‐phase bilayer an excellent candidate for low‐loss electronics or plasmonics and hot electron utilization. Our findings pave the way for advanced hetero‐structure fabrication with metal oxides and provide a novel platform for fundamental research and practical applications in renewable solar energy, integrated photonics, spintronics, quantum information, and other fields.

## 4 Experimental Section

### Fabrication of TiO_2_ Epitaxial Films

Using a magnetron sputtering system with a high‐purity TiO_2_ ceramic target (>99.99%), TiO_2_ thin films were deposited on LaAlO_3_ (LAO) substrates (<001>, 10 × 10 × 1 mm^3^, Hefei CPI Equipment & Technology Co., Ltd). Before the deposition process, the substrates were subjected to a sequential ultrasonic cleaning in acetone, alcohol, and deionized water for 10 min each step, and were then dried by nitrogen flow. The chamber was evacuated to a background pressure below 4.5 × 10^−6^ Torr, and high‐purity argon (Ar, 10 standard cubic centimeters per minute (sccm)) was introduced into the chamber. A throttle valve was used to control the growth pressure at 1 × 10^−3^ Torr. A capacitively coupled RF plasma with a power of 120 W was applied to sputter the TiO_2_ target. The substrate stage was rotated at a speed of 10 rpm. A varied‐angle spectroscopic ellipsometer at three incident angles of 65°, 70°, and 75° (ELLIP‐SR‐II, Shanghai Bright Enterprise Development Co.) was employed to calibrate the growth rate. The as‐deposited films were annealed in a tube furnace (MTI, OTF‐1200X) at atmospheric pressure in the air. The samples were heated from room temperature to 550 °C for 10 min and held at this temperature for 2 h. Subsequently, the samples were cooled down from 550 to 200 °C in 2 h and then down to room temperature with furnace cooling.

### Fabrication of TiO_2_/Ti_3_O_4_ Hetero‐Phase Bilayer

The TiO_2_ epitaxial films were placed in a quartz‐tube reactor (I.D. 50 mm) with a water‐cooled induction copper coil outside, which was then evacuated to 60 mTorr. Then Ar (20 sccm) and H_2_ (2 sccm) were introduced into the reactor, and the pressure was raised to 330 mTorr. A plasma glow ball was initiated by applying an inductively coupled RF power of 400 W and appropriately tuning the matching network. The plasma was sustained for the desired duration (i.e., 10, 20, 30, and 40 s).

### Material Characterization

Cross‐sectional samples for microstructure analysis were prepared by ion polishing using a Thermo Fisher Helio Nanolab G3 UC. STEM‐HAADF was conducted on a Thermo Fisher Titan Themis G2 60–300 operating at 200 kV. EELS was performed on a Gatan Quantum 965 GIF system. Dual EELS data were obtained with a spectrometer dispersion chosen for simultaneous visualization of both zero‐loss and core‐loss (O K and Co L edges). The energy resolution determined by full‐width at half‐maximum of the zero‐loss peak was ≈1.2 eV. Energy dispersion of 0.25 eV/channel and instantaneous dwell time of 0.5 s were used to probe the valence states. SAED was conducted on an FEI Talos F200X. The structure of the hetero‐phase bilayer was investigated by HRXRD using a Rigaku Smart Lab 3 kW. XRR was carried out on a PANalytical EMPYREAN. Ultraviolet Photoelectron Spectroscopy was performed using a PHI 5000 VersaProbe III with He I source (21.22 eV) under an applied bias of −9.0 V.

### Charge Transport Investigation

The temperature‐dependent electrical resistivity and Hall Effect measurements were conducted using an Ecopia HMS‐5000 with a magnetic field of 0.553 T. The room‐temperature electrical resistivity and Hall Effect measurements were performed using an MMR K2500‐RTSL, MK50 with a magnetic field of 0.845 T. To enhance the electrical contact, indium soldering was utilized to bond the wires and film surfaces.

## Conflict of Interest

The authors declare no conflict of interest.

## Supporting information

Supporting Information

## Data Availability

The data that support the findings of this study are available from the corresponding author upon reasonable request.
