# Supplementary material for: Plasma‐Induced 2D Electron Transport at Hetero‐Phase Titanium Oxide Interface
Source: Adv Sci (Weinh). 2023 Dec 7;11(5):2304919. doi: 10.1002/advs.202304919 (PMC10837385; doi:10.1002/advs.202304919)
Supplement: Supplementary file 1 — Supporting Information [file ADVS-11-2304919-s001.pdf]

ADVANCED SCIENCE
Open Access

Supporting Information

for *Adv. Sci.*, DOI 10.1002/advs.202304919

Plasma-Induced 2D Electron Transport at Hetero-Phase Titanium Oxide Interface

Kehan Yu, Xinglong Li, Haoyu Zhao, Chen Ma, Zhongyue Wang, Peng Lv, Ertao Hu*, Jiajin Zheng, Wei Wei and Kostya (Ken) Ostrikov*

Supporting Information

Plasma-Induced Two-Dimensional Electron Transport at Hetero-Phase Titanium Oxide Interface

Kehan Yu, Xinglong Li, Haoyu Zhao, Chen Ma, Zhongyue Wang, Peng Lv, Ertao Hu*, Jiajin Zheng, Wei Wei, and Kostya (Ken) Ostrikov*

Prof. K. Yu, X. Li, H, Zhao, C. Ma, Dr. Z. Wang, Dr. P. Lv, Dr. E. Hu, Dr. J. Zheng, Prof. W. Wei
College of Electronic and Optical Engineering & College of Flexible Electronics (Future Technology), Nanjing University of Posts and Telecommunications, Nanjing 210023, China
E-mail: kehanyu@njupt.edu.cn, iamethu@njupt.edu.cn

Prof. K. Yu, Dr. J. Zheng, Prof. W. Wei
Jiangsu Province Engineering Research Center for Fabrication and Application of Special Optical Fiber Materials and Devices, Nanjing 210036, China

Prof. K. Ostrikov
School of Chemistry and Physics and QUT Centre for Materials Science, Queensland University of Technology (QUT), Brisbane, QLD 4000, Australia

Atomic force microscopy measurements

The surface morphologies of the LAO substrate and the TiO_2 layers were characterized by atomic force microscopy (AFM, FSM-Nanoview 1000 AFM), as depicted in Fig. S1. The LAO substrate exhibited an atomically smooth single-crystalline surface with a root-mean-squared roughness (Sq) of 1.3 Å (Fig. S1A). The Sq increased to 5.1 Å after the sputtering of 50 nm-thick TiO_2 (Fig. S1B), and further increased to 1.4 nm after post-annealing (Fig. S1C). These results indicate that the post-annealing facilitated the crystallization of TiO_2.

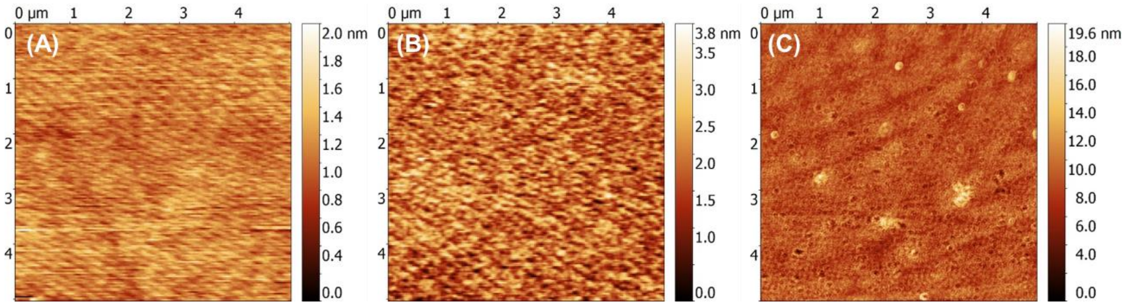

Figure S1. Surface morphologies of (A) (001) plane of LAO single crystal, (B) TiO_2 film sputtered on the LAO substrate, and (C) the sputtered TiO_2 film after post annealing.

Mechanism of plasma induced phase transition

There are two key elements to successfully preparing TiO_2/Ti_3O_4 hetero-phase bilayer using PIPT: a dazzling glow ball and the addition of a small amount of hydrogen. This differs from previous reports and implies different mechanisms. The dazzling glow ball in PIPT should belong to a type of striation (Fig. 1D), representing the resonant absorption of RF electromagnetic waves by ionized gas, so the energy density within the glow ball will greatly exceed that of a typical diffuse glow discharge. In contrast, diffuse plasmas cannot produce Ti_3O_4 even after 30 min of treatment in our experiment. Once the plasma creates Ti^{3+} and oxygen vacancies on the surface, the resulting free electrons will interact with the RF electromagnetic waves, producing a localized heating. This heating effect may accelerate the phase transition of TiO_2 to Ti_3O_4 in the lower layer, creating more free electrons which generate more heat. Anion vacancy-type metal oxides (e.g. TiO_2) always have some equilibrium oxygen vacancies, the concentration of which can be controlled by adjusting the temperature and oxygen partial pressure during the synthesis process.[1] Oxygen vacancies and Ti^{3+} in TiO_2 can be achieved through hydrogenation during heat treatment.[2] Hydrogen can stabilize dangling bonds; otherwise, treated TiO_2 would rapidly fade under ambient conditions. The previously reported low-temperature plasma treatments typically require additional heating from 200 to 500°C with the durations ranging from 10 min to 8 h depending on many process- and materials-related factors.[3-5] Prolonged heating leads to the uniform distribution of oxygen vacancies across entire samples, making it difficult to form heterojunctions on the nanoscale. Dilute plasma treatment only affects the surface of TiO_2 and cannot form heterojunctions either.[3-5]

The lattice mismatch between the Ti_3O_4 and TiO_2 phases induces strain in both layers. As proposed by the Materials Project, the Ti_3O_4 is in the tetragonal I4/mmm space group, and has $a = b = 0.413$ nm and $c = 0.818$ nm,[6] very close to that of the anatase TiO_2. There is still a 10% of lattice mismatching between the 2 layers. The relaxed state of TiO_2 has a $d_{(100)}$ spacing of 0.378 nm, whereas the relaxed

state of Ti_3O_4 has a $d_{(100)}$ spacing of 0.413 nm. Consequently, the TiO_2 layer experiences tensile stress along the [100] direction, resulting in a slight upward curvature and an expansion of its upper half $d_{(100)}$ spacing to 0.385 nm. Conversely, the Ti_3O_4 layer undergoes compressive stress along the [100] direction, leading to a reduction of its $d_{(200)}$ spacing to 0.193 nm, as indicated in Fig. 1H.

Comparison of the measured and predicted diffraction patterns

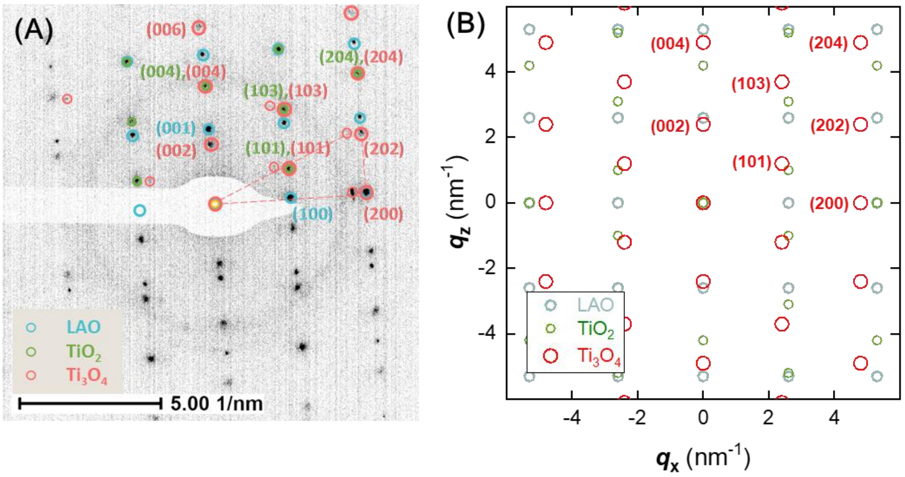

Figure S2. (A) SAED pattern of the TiO_2/Ti_3O_4 hetero-phase bilayer, taken along the [100] direction. Diffraction spots from the LAO substrate, TiO_2, and Ti_3O_4 films are indicated by colored circles and indices. (B) The predicted diffraction spots of Ti_3O_4 are plotted together with those of LAO and anatase TiO_2.

Thickness and uniformity of the TiO_2/Ti_3O_4 film

The thickness of the Ti_3O_4 layer is evaluated by 3 ways, a) ~27 nm as fitting the XRR data (Fig. S3A), b) 25 nm as exhibited by TEM images taken over a 5-µm zone (Fig. S3B-E), c) 25 nm as shown by STEM images (Fig. 1). The uniformity of the thickness of the Ti_3O_4 layer is verified by the van der Pauw measurement, in which a uniform film is mandatory. Otherwise, the measured Hall voltages would be in significant error when flipping the polarity of the magnetic field. In another word, the uniformity of the thickness of a film can be guaranteed as long as a reliable mobility is measured with the van der Pauw method.

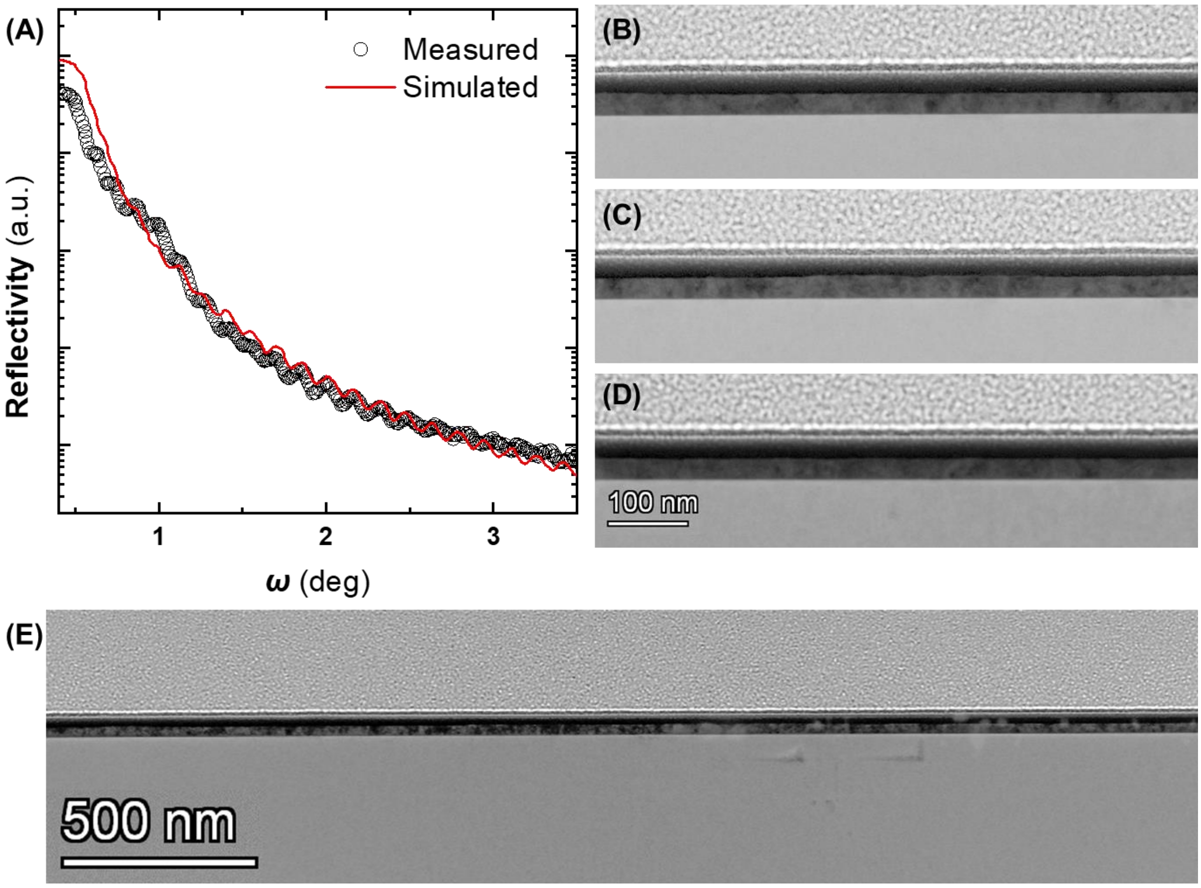

Figure S3. (A) XRR spectrum of the TiO_2/Ti_3O_4 hetero-phase bilayer. (B-E) TEM images of the TiO_2/Ti_3O_4 film taken over a 5-µm zone from the sample.

Table S1. Fitting results of the XRR the TiO_2/Ti_3O_4 film.

Layer	Layer Description	Density (g/cm^3)	Thickness (nm)
Substrate	$LaAlO_3$	6.52	6E5
1, 0	TiO_2, anatase	3.834	26.037
2, 0	Ti_3O_4	3.175	28.639

Linear combination and linear least-square fitting method

First, the intensities of Ti-$L_{2,3}$ edges in the EELS spectra were normalized within energy window 453 – 470 eV. Then, a linear combination of the EELS spectra of pure TiO_2 and Ti_2O_3 for Ti-$L_{2,3}$ edges was conducted with linear least-square fitting. Considering the quite similar EELS spectra for Ti^{2+} and Ti^{3+}, especially at the Ti-$L_{2,3}$ edge, it is reasonable to take the Ti_2O_3 as a reference.[7] The Ti^{2+} and Ti^{3+} can be treated as a whole. The weight of the combination is the fraction of Ti^{3+} (Ti^{2+}) and Ti^{4+} ions. The EELS spectrum of Ti^{3+} (Ti^{2+}) was collected from commercial trigonal Ti_2O_3 with $R\bar{3}c$ corundum structure (Sigma-Aldrich, 99.99%), because commercial tetragonal Ti_3O_4 is currently unavailable. The EELS spectrum of Ti^{4+} ions originated from laboratory sputter-deposited anatase TiO_2.

Fitting confidence of the sheet resistance vs. temperature

Since the *e-e* interaction mechanism takes effect over the whole temperature range, the resistance dependence on ln$T$ and T^2 is effective all the way above 90 K. The fitting result using the formula $R_{SH} = R_0 + R_1T^2 + R_2T^5 + R_3\,ln\frac{1}{T}$ (Eq. 1 in the manuscript) shown in **Figure S4** presents a very low Chi square and a close-to-one coefficient of determination (R square), indicating a properly used fitting model and successful fitting.

	\i(R)\-(SH)
Number of Points	20
Degrees of Freedom	16
Reduced Chi-Sqr	2.40227E-5
Residual Sum of Squares	3.84364E-4
R-Square (COD)	0.99967
Adj. R-Square	0.9996
Fit Status	Succeeded(100)

Fit Status Code :
100 : Fit converged. Chi-Sqr tolerance value of 1E-9 was reached.

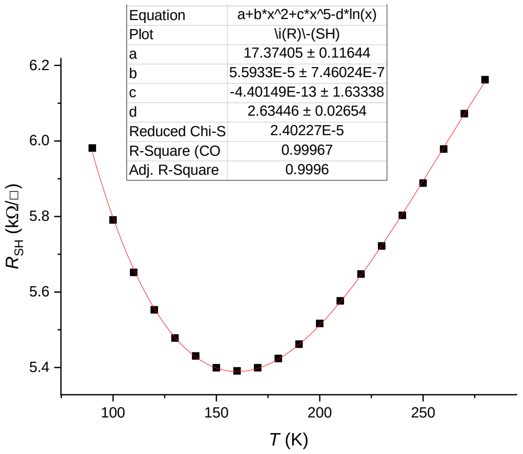

Figure S4. Fitting results of the sheet resistance vs. temperature.

Furthermore, when subtracting the residual resistance R_0, phonon scattering term (T^5), and T^2 term from the resistance, i.e.

$$\Delta R(T) = R_{SH} - (R_0 + R_1T^2 + R_2T^5)$$

We thus found a perfect linear dependence of $\Delta R(T)$ on ln$T$, as shown in Fig. 4D in the manuscript, which is also shown below (**Figure S5**). We further note that the relationship between the corrections to conductivity $\Delta\sigma$ and resistance ΔR is $\Delta\sigma = \Delta\left(\frac{1}{R}\right) = -\left(\frac{\Delta R}{R_0^2}\right)/\left(\frac{e^2}{\pi h}\right)$.

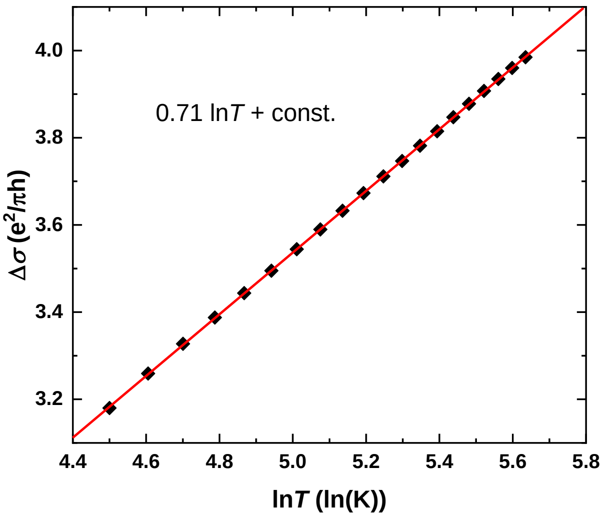

Figure S5. The correction to the conductivity of a 2D Fermi liquid system linearly fitted with ln$T$ expressed in quantum conductance ($\frac{e^2}{\pi h}$).

Overall, although there are only 7 data points below 160 K, the fitting result to 20 points over the full temperature range is still very reliable.

Table S2. Fitting parameters of Eq. (1).

R_0 (kΩ)	R_1 (kΩ K^{-2})	R_2 (kΩ K^{-5})	R_3 (kΩ)
17.37	5.59×10^{-5}	-4.40×10^{-13}	2.63

2D versus potential 3D transport in TiO_2/Ti_3O_4

In theory, the resistance (or conductance) dependences on temperature for different localization and dimensions, which are summarized in literature,[8] are listed in **Table S2**.

Table S3. Resistance (or conductance) dependences on temperature for different localization and dimensions.

Weak localization

	1D	2D	3D
$\Delta\boldsymbol{\sigma}(\boldsymbol{T}) \propto$	$-T^{-p/2}$	$p\ ln\,T$	$T^{p/2}$
$\Delta\boldsymbol{R}(\boldsymbol{T}) \propto$	$T^{-p/2}$	$-p\ ln\,T$	$-T^{p/2}$

Strong localization

<table>
<tr><td></td><td colspan="3">$T < T_0$: $\sigma(T) \propto e^{-c\left(\frac{T_0}{T}\right)^{\beta}}$
$R(T) \propto e^{c(T_0/T)^{\beta}}$</td><td>$T > T_0$</td></tr>
<tr><td rowspan="2">$\boldsymbol{\beta} =$</td><td>1D</td><td>2D</td><td>3D</td><td rowspan="2">$\sigma(T) \propto e^{-\frac{E_C-E_F}{k_B T}}$
$R(T) \propto e^{\frac{E_C-E_F}{k_B T}}$</td></tr>
<tr><td>1/2</td><td>1/3</td><td>1/4</td></tr>
</table>

In experiment, the dependence of sheet resistance on temperature was first measured to obtain the logarithmic dependence (ln$T$). Then, applying the Ioffe-Regel criterion $k_F l$ = 4.07 >>1, one can ascribe the electron transport of the sample to the **weak localization** regime. According to the theory described in the Ref. 8, also summarized in Table S2, the electron transport is **2D**. In addition, it is also verified the electron transfer and interface electron accumulation of the TiO_2/Ti_3O_4 heterostructure through the EELS scanning (Section of Electron transfer across the heterointerface in the main text), which assisted in explaining the 2D transport of electrons at the TiO_2/Ti_3O_4 interface.

Finally, the fundamentally different electric properties of TiO_2/Ti_3O_4 and Ti_3O_4 can differentiate the

transport properties. The high mobility (around 10 $cm^2V^{-1}s^{-1}$) is only possible for crystalline TiO_2 according to the data in literature, and the mobilities of amorphous and defective titanium oxide are at least one order of magnitude lower. In **Figure S6**, we compare the electrical properties (μ vs n) of TiO_2/Ti_3O_4 and reported TiO_2 materials. Obviously, that the mobility reported in this work is comparable to the mobility of crystalline TiO_2 and one order of magnitude higher than that of amorphous TiO_2. Moreover, The Ti_3O_4 has an essentially different electrical transport mechanism from the TiO_2/Ti_3O_4 hetero-phase bilayer. As shown in **Figure S7**, the resistances of them have the opposite dependence with temperature. Above room temperature, the TiO_2/Ti_3O_4 still behaves as a 2DEL, but the Ti_3O_4 behaves like a semiconductor.

Overall, it is concluded that the 2D transport is indeed the dominant transport mechanism in TiO_2/Ti_3O_4.

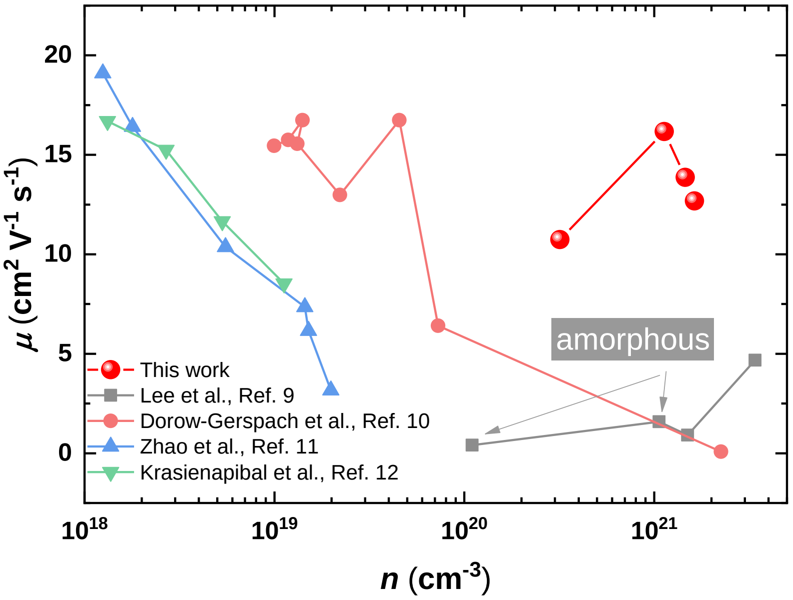

Figure S6. Dependence of electron mobility on bulk concentration of TiO_2/Ti_3O_4 and reported TiO_2 materials. Data are extracted from Ref. [9-12].

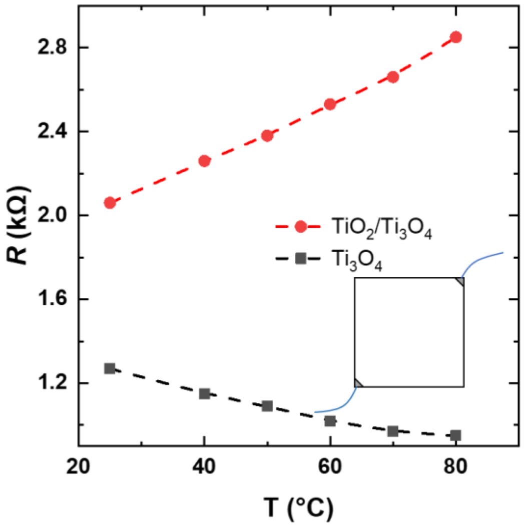

Figure S7. Comparison of the resistance (measured across the diagonal points of the samples, shown in the inset) dependences on temperature of **TiO_2/Ti_3O_4** and pure Ti_3O_4.

Why there is no short circuit by the Ti_3O_4 layer in the overall conduction:

The resistance of Ti_3O_4 decreases with increasing temperature, and the same is true for γ-Ti_2O_3, which is just the opposite for TiO_2/Ti_3O_4. If the Ti_3O_4 (a semiconductor) participates in conduction, the resistance temperature dependence above 160 K will be more complicated than the formula:

$$R_{SH} = R_0 + R_1T^2 + R_2T^5 + R_3\,ln\frac{1}{T}$$

A component of exponential decrease with temperature should be involved at least. Therefore, it is reasonable to conclude that the Ti_3O_4 layer does not participate in conduction, and the short circuit is unlikely to occur. The reason might be depletion of Ti_3O_4 in the heterostructure, despite itself is conductivity.

UPS for work function of TiO_2/Ti_3O_4

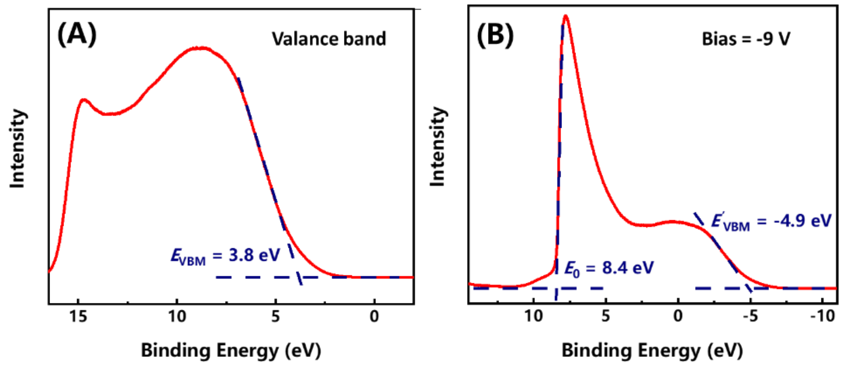

Figure S8. Ultraviolet photoelectron spectroscopy (UPS) of TiO_2/Ti_3O_4 hetero-phase bilayer without (A) and with (B) a bias of -9 V.

Mean free path of electrons in the 2DEL

Mean free path

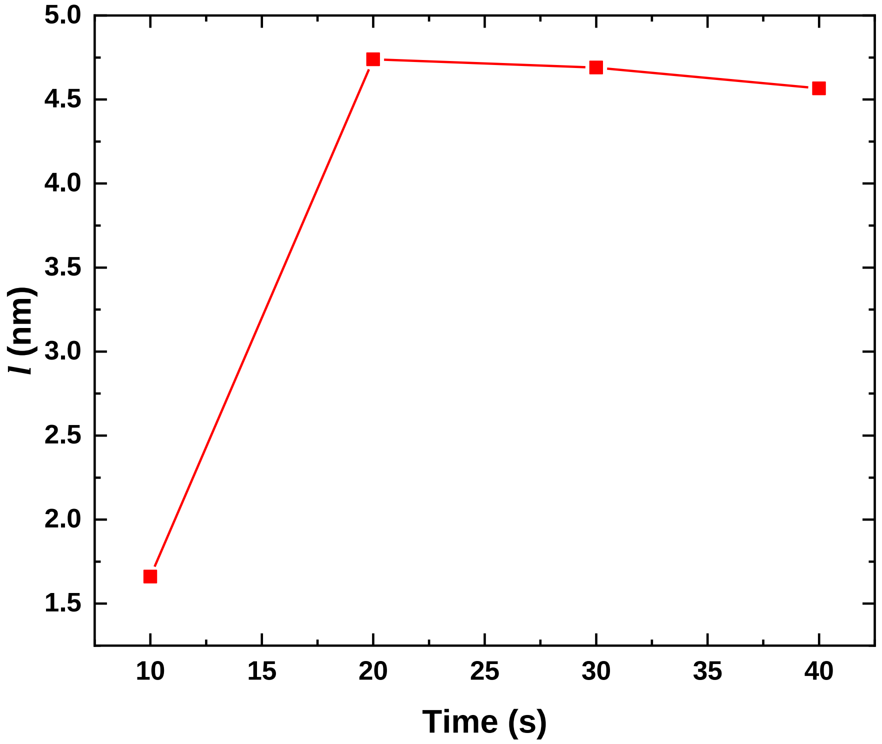

Figure S9. The variation of mean free path *l* obtained for plasma treatment from 10 to 40 s.

Supplementary calculation details

Ioffe-Regel criterion of weak localization: $k_F l >> 1$

$$k_{\mathrm{F}} l = \frac{h}{e^2}(R_{\mathrm{SH}})^{-1} = \frac{25.8\ \mathrm{k\Omega}}{R_{\mathrm{SH}}}$$

Drude conductivity: $\sigma = \frac{e^2}{\hbar} \cdot \frac{N}{k_{\mathrm{F}}^2} \cdot k_{\mathrm{F}} l$

In a 2D structure: $k_{\mathrm{F}}^2 = 2\pi N$, $\frac{N}{k_{\mathrm{F}}^2} = (2\pi)^{-1}$

$$\sigma = \frac{e^2}{\hbar} \cdot \frac{1}{2\pi} \cdot k_{\mathrm{F}} l = \frac{e^2}{h} k_{\mathrm{F}} l$$

Calculation of mean free path l:

In a 2D structure: $k_{\mathrm{F}}^2 = 2\pi N$, $k_{\mathrm{F}} = \sqrt{2\pi N}$,

$$l = \frac{k_{\mathrm{F}} l}{\sqrt{2\pi N}} = \frac{25.8\ \mathrm{k\Omega}}{R_{\mathrm{SH}}\sqrt{2\pi N}}$$

References

(1) Agrawal, A.; Cho, S. H.; Zandi, O.; Ghosh, S.; Johns, R. W.; Milliron, D. J., Localized Surface Plasmon Resonance in Semiconductor Nanocrystals. *Chem. Rev.* **2018,** *118*, 3121-3207. https://doi.org/10.1021/acs.chemrev.7b00613

(2) Chen, X.; Liu, L.; Yu, P. Y.; Mao, S. S., Increasing Solar Absorption for Photocatalysis with Black Hydrogenated Titanium Dioxide Nanocrystals. *Science* **2011,** *331*, 746-750. https://www.science.org/doi/abs/10.1126/science.1200448

(3) Koji, T.; Isao, N.; Osamu, M.; Shinichi, S.; Masazumi, A.; Tatsuhiko, I., Preparation of Visible-Light-Responsive Titanium Oxide Photocatalysts by Plasma Treatment. *Chem. Lett.* **2000,** *29*, 1354-1355. https://www.journal.csj.jp/doi/abs/10.1246/cl.2000.1354

(4) Ihara, T.; Miyoshi, M.; Ando, M.; Sugihara, S.; Iriyama, Y., Preparation of a visible-light-active TiO_2 photocatalyst by RF plasma treatment. *J. Mater. Sci.* **2001,** *36*, 4201-4207. https://doi.org/10.1023/A:1017929207882

(5) Wang, Z.; Yang, C.; Lin, T.; Yin, H.; Chen, P.; Wan, D.; Xu, F.; Huang, F.; Lin, J.; Xie, X., et al., H-Doped Black Titania with Very High Solar Absorption and Excellent Photocatalysis Enhanced by Localized Surface Plasmon Resonance. *Adv. Funct. Mater.* **2013,** *23*, 5444-5450. https://onlinelibrary.wiley.com/doi/abs/10.1002/adfm.201300486

(6) Jain, A.; Ong, S. P.; Hautier, G.; Chen, W.; Richards, W. D.; Dacek, S.; Cholia, S.; Gunter, D.; Skinner, D.; Ceder, G., et al., Commentary: The Materials Project: A materials genome approach to accelerating materials innovation. *APL Mater.* **2013,** *1*, 011002. https://doi.org/10.1063/1.4812323

(7) Li, Y.; Wang, Q.; An, M.; Li, K.; Wehbe, N.; Zhang, Q.; Dong, S.; Wu, T., Nanoscale Chemical and Valence Evolution at the Metal/Oxide Interface: A Case Study of Ti/SrTiO3. *Adv. Mater. Interfaces* **2016,** *3*, 1600201. https://onlinelibrary.wiley.com/doi/abs/10.1002/admi.201600201

(8) Lee, P. A.; Ramakrishnan, T. V., Disordered electronic systems. *Reviews of Modern Physics* **1985,** *57*, 287-337. https://link.aps.org/doi/10.1103/RevModPhys.57.287

(9) Lee, H. J.; Moon, T.; An, C. H.; Hwang, C. S., 2D Electron Gas at the Interface of Atomic-Layer-Deposited Al2O3/TiO2 on SrTiO3 Single Crystal Substrate. *Advanced Electronic Materials* **2019,** *5*, 1800527. https://onlinelibrary.wiley.com/doi/abs/10.1002/aelm.201800527

(10) Dorow-Gerspach, D.; Wuttig, M., Metal-like conductivity in undoped TiO2-x: Understanding an unconventional transparent conducting oxide. *Thin Solid Films* **2019,** *669*, 1-7. https://www.sciencedirect.com/science/article/pii/S0040609018307028

(11) Zhao, W.; Luan, C.; Ma, X.; Feng, X.; He, L.; Ma, J., Characterization of niobium-doped titania epitaxial films deposited by metalorganic chemical vapor deposition. *Materials Characterization* **2018,** *137*, 263-268. https://www.sciencedirect.com/science/article/pii/S1044580317327420

(12) Krasienapibal, T. S.; Fukumura, T.; Hirose, Y.; Hasegawa, T., Improved room temperature electron mobility in self-buffered anatase TiO2 epitaxial thin film grown at low temperature. *Japanese Journal of Applied Physics* **2014,** *53*, 090305. https://dx.doi.org/10.7567/JJAP.53.090305
